# A Dynamic Succession-Based Life-Cycle Simulation Model for Projecting Carbon Source–Sink Transitions in Urban Plant Communities

**DOI:** 10.3390/biology15131072

**Published:** 2026-07-04

**Authors:** Xiaxi Liuyang, Jiayu Lu, Yang Cao

**Affiliations:** 1 School of Human Settlements, North China University of Water Resources and Electric Power, Zhengzhou 450046, China; lyxx7776@tju.edu.cn; 2 School of Architecture, Tianjin University, Tianjin 300072, China; 3 College of Landscape Architecture and Art, Henan Agricultural University, Zhengzhou 450002, China; cao0019@henau.edu.cn

**Keywords:** plant community, carbon emissions, carbon sequestration, life cycle assessment, succession simulation model

## Abstract

Urban plant communities are widely regarded as important nature-based solutions for climate mitigation, yet their actual carbon benefits remain uncertain: vegetation growth is accompanied by carbon emissions from construction and long-term maintenance, and existing assessments rarely integrate community succession, interspecific competition, and maintenance-related emissions within a consistent life-cycle framework. To address these limitations, this study developed a dynamic succession-based life-cycle simulation model to project the 50-year carbon source–sink transitions of 150 typical urban plant communities in Tianjin, China. The model updates plant structural attributes—diameter at breast height, crown width, and tree height—iteratively by linking individual plant growth to environmental suitability and neighborhood competition through a Plant Health Index. Simulated structural trajectories were coupled with biomass equations and carbon content coefficients to estimate aboveground carbon sequestration, while construction and maintenance emissions were quantified using life cycle assessment, enabling evaluation of modeled net carbon balance rather than gross carbon sequestration alone. Under the modeled 50-year scenario, most communities were projected to act as carbon sources during the early stage but gradually shifted toward carbon sinks as biomass accumulated; 86.1% of the communities were projected to become net carbon sinks after 50 years (a scenario-based projection under specified growth, maintenance, and emission assumptions). The highest modeled net carbon balance reached 3186.08 kg·C·ha^−1^, whereas the weakest community remained a slight carbon source at −81.21 kg·C·ha^−1^. Vertical structural complexity and species richness were the strongest positive predictors of modeled net carbon balance, followed by three-dimensional green quantity and canopy closure. Among maintenance processes, fertilization was the dominant emission source, followed by pesticide application and irrigation; comparative scenario analysis showed that resource-saving maintenance consistently improved projected net carbon balance relative to high-maintenance management. These results suggest that low-carbon planting design should prioritize locally adapted species, multi-layered vertical structures, and adaptive maintenance over simply maximizing planting density or minimizing inputs. The results represent scenario-based projections of aboveground vegetation carbon balance; belowground biomass, soil carbon, litter carbon, dead organic matter, and parameter uncertainty were not fully incorporated, and future studies should address these limitations to improve the robustness and transferability of the proposed framework.

## Simple Summary

Urban green spaces are widely assumed to absorb carbon and help cities address climate change, but planting and maintaining vegetation also generate emissions through construction, irrigation, fertilization, and pesticide use. We developed a simulation model tracking how 150 urban plant communities in Tianjin, China, grow and are managed over 50 years. Most communities began as net carbon emitters but shifted to net carbon absorbers as plants matured, with 86.1% projected to achieve a positive modeled carbon balance by year 50 under the baseline scenario. Communities with multiple vegetation layers, greater species diversity, and locally adapted species performed best. These findings offer practical guidance for designing urban green spaces that deliver genuine long-term climate benefits.

## 1. Introduction

With accelerating urbanization and increasing pressure to mitigate climate change, urban green spaces (UGSs) have been widely recognized as an important component of urban green infrastructure. Beyond their recreational, aesthetic, and social functions, UGSs provide multiple ecosystem services, among which carbon sequestration has received growing attention [1]. Plant communities are the basic design and functional units through which urban green spaces deliver carbon-related benefits. Through photosynthesis and biomass accumulation, plant communities can remove atmospheric carbon dioxide and store carbon in woody tissues, leaves, and other aboveground components. Therefore, optimizing plant community structure and composition has become an important pathway for improving the carbon mitigation capacity of cities.

However, the carbon sink function of urban plant communities is more uncertain than is often assumed. Unlike natural plant communities, urban artificial plant communities are usually established and managed to meet multiple functional objectives, including visual quality, shading, recreational, safety, and spatial order. Species selection and planting design often emphasize ornamental and functional requirements, whereas long-term ecological adaptability and interspecific relationships may receive less attention. As a result, species with similar ecological niches may be planted at high densities in limited spaces, intensifying competition for light, water, nutrients, and growing space. Such competition may suppress individual growth, constrain canopy expansion, and ultimately weaken biomass accumulation and carbon sequestration capacity [2,3]. In addition, urban plant communities require continuous management, including irrigation, fertilization, pesticide application, pruning, replanting, and litter disposal. These practices consume energy and materials and generate carbon emissions [4]. Urban plant communities should therefore not be regarded as automatic or continuous carbon sinks; their net carbon balance depends on the dynamic relationship between biomass accumulation and construction- and maintenance-related emissions.

Urban forest carbon accounting has been extensively investigated across multiple regions. In North America, Nowak [5] quantified carbon storage and annual sequestration for urban trees across the United States using field inventories and allometric equations. In Europe, Strohbach [6] applied life-cycle assessment to urban green spaces in Leipzig and found that construction and maintenance emissions could substantially offset vegetation carbon sequestration, particularly for intensively managed sites. In East Asia, a growing body of studies has examined urban forest carbon dynamics in the context of rapid urbanization and high planting densities [7]. Among the tools developed to support these assessments, i-Tree Eco, UFORE, CITYgreen [8,9], and related urban forestry models have provided important methods for estimating tree-level and city-scale carbon storage and sequestration. These studies have laid a strong foundation for incorporating urban vegetation into climate-mitigation strategies and green infrastructure planning [10,11]. Nevertheless, most established tools are primarily designed for tree inventories or landscape-scale assessment and are less suited to simulating the life-cycle carbon source–sink trajectories of designed plant communities as planting units.

At the plant-community scale, empirical studies have shown that carbon storage and sequestration are strongly shaped by vegetation composition and structural configuration [12]. Mixed broad-leaved communities, uneven-aged stands, and multi-layered plant communities generally store more carbon than simplified or single-layer shrub-dominated communities [13]. Structural attributes such as planting density, canopy closure, three-dimensional green quantity, tree-shrub ratio, species richness, and vertical stratification have also been reported to influence community carbon storage and sequestration [14,15,16,17]. However, most existing community-scale studies still rely on static carbon-stock estimation or short-term field measurements. Such approaches are useful for describing current carbon storage, but they cannot reveal how plant communities develop over time, how competition alters growth trajectories, or when a community may shift from a net carbon source to a net carbon sink.

Designed urban plant communities are dynamic systems. During their life cycle, individual plants grow, compete, decline, or die, while the overall community structure changes accordingly. These successional processes affect carbon sequestration by altering biomass, canopy structure, light interception, and spatial resource allocation. Process-based and individual-based ecological models have long been used to simulate forest growth, succession, competition, establishment, and mortality [18]. These models show that long-term biomass accumulation is shaped by species traits, environmental conditions, resource availability, neighborhood interactions, and successional stage [19,20,21]. However, such ecological processes are rarely coupled with life-cycle carbon accounting in the assessment of designed urban plant communities, and when succession and competition are ignored, the long-term carbon sink potential of urban plant communities may be systematically overestimated or underestimated.

A further limitation is that carbon emissions generated during construction and maintenance are insufficiently integrated with plant growth simulation in most existing frameworks [22]. From a life-cycle perspective, urban plant communities may shift dynamically between carbon sources and carbon sinks over time. Emissions can arise from seedling transportation, planting operations, irrigation, fertilization, pesticide application, pruning, litter disposal, machinery use, and other maintenance activities. Previous studies have shown that these emissions can substantially delay the time required for urban green spaces to achieve carbon neutrality. For example, Zhang et al. found that 40 years were required to achieve carbon balance, with transportation distance and landscape material selection identified as key limiting factors [23]. Lin et al. estimated the 50-year life-cycle carbon source and sink of the Longhu Outer Ring urban green space in Zhengzhou and found that carbon balance could only be achieved after 20 years, mainly due to material input and maintenance-related emissions [24]. A 50-year simulation of urban green plant communities in Tianjin further showed that 64% of carbon emissions originated from irrigation and agrochemical application [25]. Similarly, Park et al. reported that irrigation accounted for 33–55% of operational carbon emissions in Korean urban green spaces [26]. These findings collectively indicate that a plant community with high biomass does not necessarily generate high net carbon benefits if it requires intensive maintenance, and that an integrated framework coupling vegetation growth with construction- and maintenance-related emissions is needed to evaluate net carbon balance.

Taken together, three methodological gaps remain in existing approaches to urban plant community carbon assessment. First, many assessments are based on static biomass or carbon-stock estimation and have limited ability to capture community structural development over time. Second, ecological processes such as growth, competition, and mortality are not always incorporated into life-cycle carbon accounting. Third, maintenance-related emissions are often assessed separately from plant growth, making it difficult to determine when and under what structural or management conditions urban plant communities are projected to shift from net carbon sources to net carbon sinks. These gaps may lead to an incomplete evaluation of the carbon benefits of designed urban plant communities that require long-term management.

The proposed model is not intended to replace established urban forest assessment tools such as i-Tree Eco, CITYgreen, or UFORE, nor does it attempt to reproduce the full complexity of process-based forest succession models. Instead, it addresses a specific application gap: the community-scale life-cycle simulation of designed urban plant communities. Compared with tree-inventory-based urban forest tools, the proposed framework treats the plant community as the basic design and assessment unit, incorporating species composition, vertical structure, planting density, canopy development, and maintenance demand. Compared with conventional life-cycle assessment studies, it links construction and maintenance emissions with simulated plant growth rather than treating them as separate accounting components. Compared with general individual-based ecological models, it is designed to support low-carbon planting design by projecting modeled net carbon balance under explicit structural and maintenance assumptions.

To address these gaps, this study develops a dynamic succession-based life-cycle simulation model for urban plant communities. The model converts carbon assessment from a static estimation of biomass carbon into a dynamic simulation of carbon source–sink trajectories over a 50-year life cycle. It incorporates environmental suitability and neighborhood competition into plant growth simulation through a Plant Health Index, allowing diameter at breast height, crown width, and tree height to be updated dynamically. It also couples succession-driven aboveground biomass carbon sequestration with carbon emissions from construction and maintenance, thereby evaluating modeled net carbon balance rather than gross carbon sequestration alone. The model outputs are scenario-based projections under specified structural, growth, and maintenance assumptions rather than direct long-term empirical observations.

Using 150 typical urban plant communities in Tianjin, China, this study applies the proposed model to address three questions:

(1) How are urban plant communities projected to shift between carbon sources and carbon sinks over a 50-year life cycle?
(2) How do simulated community succession, neighborhood competition, and maintenance emissions jointly affect modeled net carbon balance?
(3) Which structural parameters and planting configurations are associated with stronger modeled carbon performance and may support low-carbon plant community design?

By answering these questions, this study seeks to move beyond static carbon-stock assessment and provide a dynamic, planning-oriented simulation framework for optimizing urban plant communities as long-term low-carbon green infrastructure.

## 2. Materials and Methods

### 2.1. Study Area, Sampling Design and Plant Community Selection

This study was conducted in the main urban area of Tianjin, China (38°34′–40°15′ N, 116°43′–118°04′ E). Tianjin is located in the eastern part of the North China Plain along the western coast of the Bohai Sea, with a warm-temperate monsoon climate characterized by a mean annual temperature of approximately 12–13 °C, a mean annual precipitation of approximately 550–600 mm concentrated mainly from June to September, and a frost-free period of approximately 200–210 days. Urban soils are strongly affected by alluvial deposition, salinization, alkalization, compaction, and human disturbance. The urban green spaces are dominated by artificially planted tree, shrub, and tree-shrub mixed communities, including common species such as *Populus tomentosa*, *Fraxinus chinensis*, *Styphnolobium japonicum*, *Platanus acerifolia*, *Salix babylonica*, *Koelreuteria paniculata*, *Buxus sinica*, *Ligustrum quihoui*, *Lonicera japonica*, and *Juniperus* species. These climatic, soil, and vegetation conditions make Tianjin suitable for evaluating the long-term modeled carbon balance of managed urban plant communities.

Sampling sites were selected from urban parks and public green spaces using a stratified systematic random sampling strategy. Stratification was based on four dimensions: (1) green-space type, including park, square, affiliated, and protective green spaces; (2) green-space age, including 0–10, 10–30, and >30 years; (3) maintenance intensity, pre-classified as recommended-, standard-, or high-maintenance; and (4) horizontal community structure, including open, semi-closed, and closed communities. Within each stratum, quadrats were randomly arranged where field conditions allowed. Site selection additionally considered park type, geographical location, green-space size, plant community structure, management intensity, vegetation characteristics, and accessibility. Only well-managed artificial plant communities with relatively stable growth conditions were included to ensure the comparability of community structure, carbon sequestration, and maintenance-related carbon emissions.

**Figure 1:**
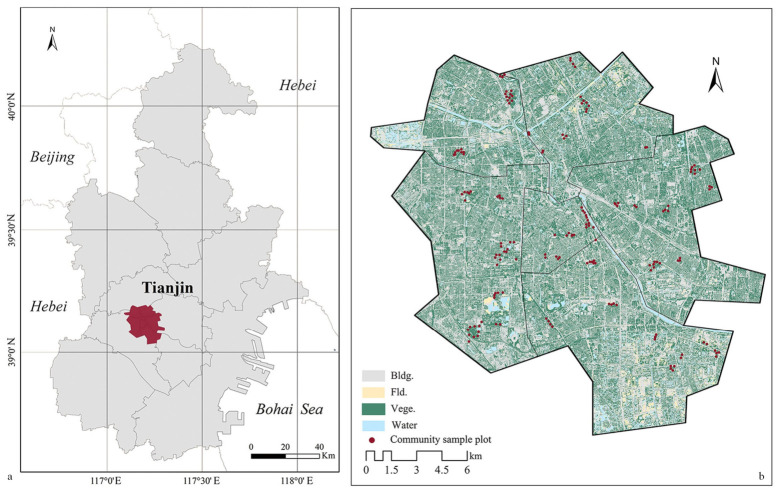
Spatial distribution of community sampling plots. (**a**): Location map of the study area; (**b**) Detailed sampling plot distribution map within central Tianjin.

A total of 30 sampling sites were selected in the main urban area of Tianjin. Within each site, standard 10 m × 10 m quadrats were arranged on a 50 m × 50 m grid wherever possible to improve spatial representativeness. When the shape or area of a green space restricted the establishment of a standard square quadrat, the quadrat shape was adjusted while maintaining a total area of 100 m^2^. In total, 150 plant community quadrats were investigated (Figure 1). All 150 quadrats were used for dynamic succession simulation, aboveground carbon sequestration accounting, life-cycle emission accounting, modeled net carbon balance calculation, structural-factor analysis, and maintenance-scenario comparison.

Field surveys were conducted once every summer from 2020 to 2025, yielding six repeated survey rounds for each quadrat. The 2020 survey served as the baseline state for model initialization for all 150 quadrats. The repeated measurements from 2021 to 2025 were used to calibrate species-specific maximum annual growth increment parameters in the logistic growth functions and to constrain the mortality threshold of the Plant Health Index. The 2021–2025 measurements were therefore used for parameter calibration and plausibility checking rather than as additional initial states for the 50-year simulation, ensuring that all 150 quadrats were initialized from a consistent baseline year.

For clearer visualization of model outputs, 36 representative communities were selected from the 150 investigated communities for display in the growth, carbon sequestration, carbon emission, and source–sink transition figures. These 36 communities were selected only for visualization purposes and were not used as a substitute for the full dataset. Selection criteria included community type, dominant growth form, horizontal structure (open/semi-closed/closed), dominant species, canopy closure, species richness, vertical structural layers, three-dimensional green quantity, maintenance level, and modeled carbon-balance performance.

**Figure 2:**
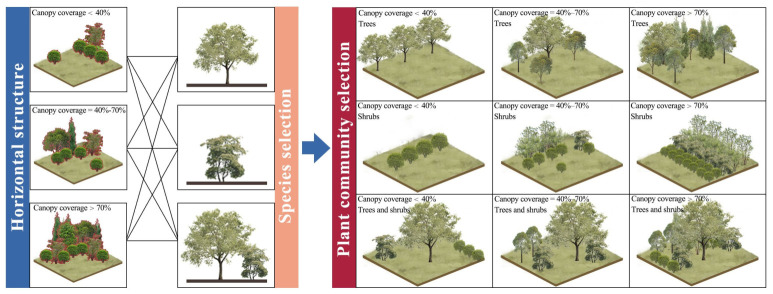
Plant community selection.

Plant communities were classified according to their horizontal structural characteristics into three types: open, semi-closed, and closed. Each structural type included communities dominated by trees, shrubs, and tree–shrub mixtures, allowing the carbon balance of different community types to be compared under a consistent life-cycle assessment framework (Figure 2).

During each survey, the structural characteristics of all woody plants within each quadrat were recorded. For each quadrat, the following information was obtained: species identity, number of individuals, growth form, diameter at breast height, plant height, crown width, canopy closure, vertical structural layers, and planting density. For trees, diameter at breast height was measured at 1.3 m above ground using a diameter tape; tree height was measured using a hypsometer (Model ECII D-R); and crown width was measured in two perpendicular directions and averaged. For shrubs, basal diameter or crown diameter, shrub height, and coverage were recorded. Canopy closure and leaf area-related parameters were measured using a YMJ-D leaf area meter (Manufactured by Zhejiang Top Cloud-Agri Technology Co., Ltd., Hangzhou, China) and an LAI-2200C Plant Canopy Analyzer (Manufactured by LI-COR, Inc., Lincoln, NE, USA). Planting density was calculated as the number of plant individuals per unit area and converted to plants ha^−1^. Vertical structural layers were identified according to the presence of tree, sub-tree, shrub, and herb layers. Three-dimensional green quantity was calculated based on plant height, crown width, and coverage using an instrument-based scanning method.

Maintenance-related data were obtained from questionnaires, work-log records, and direct interviews with green-space managers, covering irrigation frequency and volume, fertilization frequency and amount, pesticide application, pruning frequency, litter disposal, machinery use, transportation distance, fuel consumption, and labor input. The structural attributes at 10, 20, 30, 40, and 50 years were simulated using the dynamic plant community succession model described in Section 2.3, rather than obtained through repeated long-term field observations.

### 2.2. Dynamic Succession-Based Life-Cycle Carbon Accounting Framework

This study developed a dynamic succession-based life-cycle carbon accounting model to evaluate the long-term carbon source–sink transitions of urban plant communities. The framework consists of three coupled modules: the dynamic succession module, which simulates changes in diameter at breast height, crown width, tree height, and plant survival over a 50-year period; the carbon sequestration module, which estimates aboveground biomass carbon at different life-cycle stages using biomass equations and carbon content coefficients; and the life-cycle emission module, which quantifies carbon emissions from construction and maintenance processes, including seedling transportation, planting operations, irrigation, fertilization, pesticide application, pruning, litter disposal, machinery use, and labor input. By linking these modules within a single consistent boundary, the framework shifts carbon assessment from a static estimation of biomass carbon stocks to a dynamic evaluation of net carbon source–sink trajectories over the full life cycle.

The framework was developed as a process-informed scenario-comparison tool rather than a deterministic prediction model. The 50-year outputs should therefore be interpreted as projections under specified assumptions about plant growth, neighbourhood competition, maintenance intensity, and emission factors.

Life cycle assessment was used to quantify the carbon balance of urban plant communities over a 50-year period, following the general logic of LCA including goal and scope definition, life cycle inventory analysis, impact assessment, and interpretation [27]. The goal was to evaluate the net carbon balance of different plant communities and identify the structural and management factors that influence their long-term carbon sink capacity [28].

A cradle-to-grave system boundary was established to account for the main carbon emissions associated with the construction and maintenance of plant communities. The 50-year time horizon was adopted because many urban trees approach relatively stable growth after several decades, while longer-term simulations would introduce greater uncertainty related to future technology, maintenance practices, and environmental change [25].

The functional unit was defined as the carbon balance of a plant community per unit area over the 50-year life cycle. Net carbon balance was calculated by comparing carbon sequestration through aboveground biomass accumulation with carbon emissions generated during construction and maintenance. Positive values indicate that the community functions as a net carbon sink, whereas negative values indicate that it functions as a net carbon source.

To improve the clarity of the methodological framework, this study followed four main steps: (1) field survey and plant community characterization, (2) dynamic succession simulation, (3) life-cycle carbon accounting, and (4) statistical analysis of structural factors and maintenance scenarios.

### 2.3. Dynamic Plant Community Succession Model

**Figure 3:**
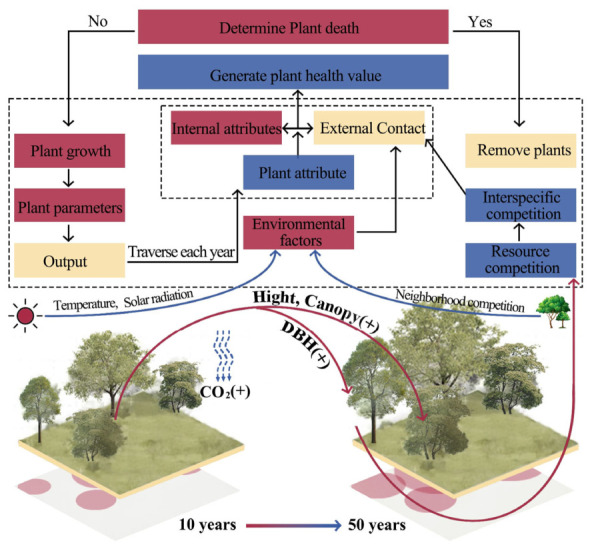
Plant growth succession model.

The dynamic succession module represents plant community succession as the cumulative outcome of individual plant growth, environmental suitability, and neighbourhood competition (Figure 3). Each plant individual was updated iteratively over the 50-year simulation period, with annual changes in diameter at breast height, crown width, and tree height used to track structural development. The core driver of this module is a Plant Health Index (PHI) [29], jointly determined by intrinsic species traits, environmental suitability, and neighbourhood competition. Rather than prescribing growth along fixed species-level curves, the module adjusts annual growth increments in proportion to PHI: when environmental conditions are suitable and competition is weak, PHI remains high and growth proceeds near the species-specific maximum; as stress or canopy overlap increases, PHI declines, reducing growth increments accordingly; plant death and removal are assumed when PHI falls below zero. As a result, simulated carbon sequestration reflects not only species-specific growth potential but also the constraining effects of community structure, spatial competition, and environmental conditions.

To improve model reproducibility, the key parameters used in the dynamic succession model are summarized in Table 1. These parameters include the temperature sensitivity coefficient (kT), neighborhood competition decay coefficient (β), coupling coefficient (α), temperature weight (wT), and health-response coefficient (kf). Parameter values were determined using a combination of published literature, existing individual-based plant growth and neighborhood competition models, and calibration with the 2020–2025 repeated field measurements. Parameters derived from field calibration were constrained by observed annual increments in diameter at breast height, crown width, and tree height, as well as observed mortality records.

The specific literature sources supporting these parameters include studies on plant temperature response, high-temperature stress, neighborhood competition, field-of-neighborhood models, multi-weight environmental stress assessment, and individual-based forest succession simulation. The final parameter values were adjusted using the repeated field measurements from 2020 to 2025 to improve consistency with local urban plant community growth patterns.

The inherent characteristics of plants serve as the fundamental basis for their health, with different species showing different levels of adaptability to environmental conditions. To describe plant responses to environmental factors, this study used suitability and stress functions to represent the effects of temperature and solar radiation on plant health. For each species, an adaptation range and a tolerance range were defined. For example, if a species grows optimally between 12 and 18 °C, its optimum temperature range is defined as [12,18]; if it can survive between −10 and 30 °C, its tolerance range is defined as [−10, 30]. For environmental factors that cannot be expressed directly by measured values, an interval within [0, 1] was used to represent the degree of suitability.

Temperature suitability. Temperature is an important environmental factor affecting plant physiological activity and growth. When annual temperature falls within the optimum range of a species, temperature stress is assumed to be minimal. When annual temperature deviates from the optimum range or approaches the tolerance boundary, temperature stress increases and plant health declines. The effect of temperature on plant health was calculated using a piecewise function:

(1) $$∆T\left(t\right)=\left\{\begin{array}{lll} e^{k_{T}{\left(\frac{{|T}_{t}-T_{o}|}{T_{r}}\right)}^{2}-1} & , & if\left|T_{t}-T_{o}\right|>T_{t}\\ 0 & , & otherwise \end{array}\right.$$

where *T_t_* is annual temperature; *T_o_* is plant optimum temperature range; *T_r_* is plant tolerance temperature range; and *k_T_* is plant sensitivity coefficient.

Solar radiation suitability. Solar radiation provides the energy required for photosynthesis and directly affects plant growth. Within a suitable range, increasing radiation can enhance photosynthesis and biomass accumulation. However, when radiation exceeds the photosynthetic saturation range, excessive radiation may reduce plant health. Therefore, the effect of solar radiation on plant health was described using a radiation response function:

(2) $$∆R_{i}\left(t\right)=\frac{R}{k+R}e^{-c({R-R_{o})}^{2}}$$

where *R* is actual solar radiation; *k* is half-saturation constant; *R_o_* is Optimal radiation intensity; and *c* is inhibition coefficient.

Neighborhood competition. Competition among individual plants is a common process in plant communities. Within a confined planting space, resource competition occurs when individual plants are close to one another and their influence zones overlap [33]. Drawing on the field-of-neighborhood model, this study used two-dimensional circular regions to represent the spatial distribution and influence area of individual plants. Competitive relationships among individuals were calculated by determining the intersections of these circular influence zones [34]. Competition was mainly represented in two aspects: light competition, which is affected by plant height, position, and canopy radius; and spatial competition, which occurs when the space required by neighboring crowns overlaps.

The total competition intensity of plant individual *i* was calculated as the linear superposition of the competitive effects of all neighboring individuals:

(3) $$C_{i}\left(t\right)=\sum_{j\neq 1}\left[\frac{H_{j}^{\alpha_{i}}}{H_{i}}\cdot \frac{A_{O}}{\pi R_{c,j}^{2}}\cdot e^{-\beta \cdot d_{ij}}\right]$$

where *C_i_*(*t*): Total competitive suppression coefficient of tree *i* at time *t*; *A_O_* is the overlapping area of two canopies; *β* is the competition decay coefficient; and *d_ij_* is the inter-plant distance; *H_i_*, *H_j_*: Height of target tree *i* and neighboring tree *j*; *R_c_*_,*j*_: Crown radius of neighboring tree *j*; *α_i_*: Height coupling exponent of tree *i*.

Plant Health Index. Plant growth dynamics are influenced by environmental suitability and neighborhood competition. To quantitatively assess plant health over a given time interval, a weighted comprehensive function was used to calculate the Plant Health Index:

(4) $$f_{i}\left(t\right)=1-w_{T}\cdot ∆T_{i}\left(t\right)\cdot (1+∆C_{i}(t){)}^{\alpha }-w_{T}\cdot ∆R_{i}\left(t\right)$$

where *w_T_* is temperature weight; *α* is coupling index; and Plant death and removal are assumed when *f_i_*(*t*) < 0.

Plant death and removal were assumed to occur when the Plant Health Index fell below zero, i.e., *f_i_*(*t*) < 0. This threshold follows the logic of individual-based forest succession models and field-of-neighborhood competition frameworks, in which a negative health value indicates that the combined effects of environmental stress and neighborhood competition exceed the assumed tolerance capacity of the plant individual. This assumption is also consistent with the ecological logic that severe and cumulative physiological stress can push plants beyond mortality thresholds. The threshold was further adjusted using observed mortality and survival records from the 2020–2025 repeated field surveys. Because mortality is a highly influential ecological process, the mortality-related assumptions were also considered in the uncertainty discussion and sensitivity analysis.

Growth update of plant structural attributes. Plant growth is a complex multidimensional process. Diameter at breast height, crown width, and tree height are key indicators for assessing tree growth status and biomass. Diameter at breast height reflects trunk thickening and material accumulation capacity. Crown width represents the horizontal space occupied by the canopy and its photosynthetic potential. Tree height reflects vertical growth ability and competitiveness for light resources. Together, these indicators provide a structural basis for estimating plant biomass and carbon sequestration.

In this study, plant growth was characterized by annual changes in diameter at breast height, crown width, and tree height. The relationship between the Plant Health Index and the growth increments of these structural attributes was described using logistic-type growth functions:

(5) $$D_{i}\left(t+1\right)=D_{i}\left(t\right)+g_{m}\left(1-\frac{D_{i}}{D_{m}}\right)\left(1-e^{-k_{f}\cdot f_{i}\left(t\right)}\right)$$

(6) $$C_{i}\left(t+1\right)=C_{i}\left(t\right)+g_{m}\left(1-\frac{C_{i}}{C_{m}}\right)\left(1-e^{-k_{f}\cdot f_{i}\left(t\right)}\right)$$

(7) $$H_{i}\left(t+1\right)=H_{i}\left(t\right)+g_{m}\left(1-\frac{H_{i}}{H_{m}}\right)\left(1-e^{-k_{f}\cdot f_{i}\left(t\right)}\right)$$

where *g_m_* is maximum annual growth increment; *k_f_* is health response coefficient; *D_i_*(*t*): DBH of the *i*-th tree in year *t*; *D_i_*(*t* + 1): Updated DBH of the *i*-th tree in year *t* + 1; *D_m_*: Species-specific maximum attainable DBH; *C_i_*(*t*): Crown width of the *i*-th tree in year *t*; *C_i_*(*t* + 1): Updated crown width of the *i*-th tree in year *t* + 1; C*_m_*: Species-specific maximum attainable crown width; *H_i_*(*t*): Height of the *i*-th tree in year *t*; *H_i_*(*t* + 1): Updated height of the *i*-th tree in year *t* + 1; *H_m_*: Species-specific maximum attainable tree height.

The logistic-type growth functions were calibrated using the 2020–2025 repeated field measurements, with the 2020 survey as the baseline state and observed annual increments from 2021 to 2025 used to constrain species-specific *g_m_* and *k_f_* values. To evaluate whether the calibrated succession module could reproduce realistic long-term structural development, an independent model validation was conducted using long-term monitoring records from Tianjin People’s Park. This validation dataset was independent from the 150 quadrats used for model application and parameter calibration. It consisted of 10 quadrats and 76 trees with continuous 30-year field monitoring records and park management statistics, including observed changes in diameter at breast height, crown width, and tree height.

For the validation procedure, the initial structural records of the 76 trees were used as model inputs, and the simulated DBH, crown width, and tree height trajectories were compared with the corresponding observed values from the long-term monitoring dataset. Model performance was evaluated using mean percentage error (MPE):

$$MPE=\frac{1}{n}\sum_{i=1}^{n}\left|\frac{P_{i}-O_{i}}{O_{i}}\right|\times 100\%$$

where *P_i_* is the predicted value, *O_i_* is the observed value, and *n* is the number of observations. The validation assessed the biological plausibility of the simulated structural growth trajectories rather than the full life-cycle carbon balance outputs, which should still be interpreted as scenario-based projections under specified assumptions.

The structural attributes at 10, 20, 30, 40, and 50 years generated by the dynamic succession model were then used as inputs for biomass estimation and carbon sequestration accounting in Section 2.4.

### 2.4. Life-Cycle Carbon Sequestration, Emissions, and Net Carbon Balance

**Figure 4:**
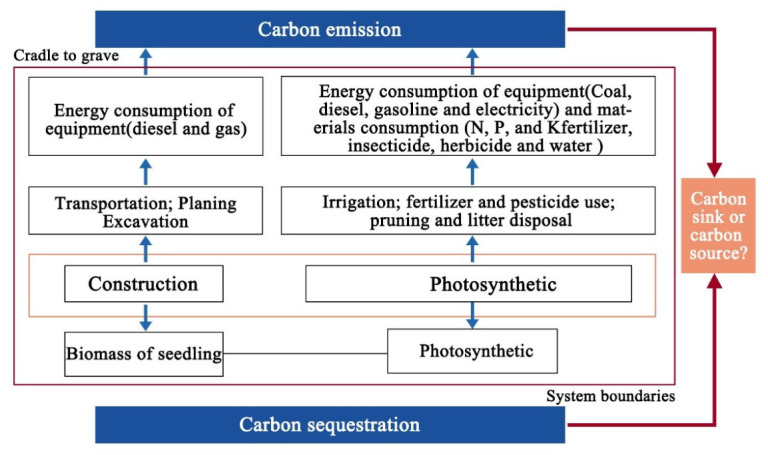
System boundaries of urban plant community.

The system boundary included the main material and energy inputs associated with the construction and maintenance of urban plant communities (Figure 4). The construction phase included tree-pit excavation, seedling transportation, and planting operations. The maintenance phase included irrigation, pruning, fertilization, pesticide application, litter disposal, and labor input. Emissions from building demolition, production of gardening tools, seedling production, seedling replacement, and maintenance after natural disasters were excluded as these fell outside the defined system boundary.

Carbon sequestration was estimated using published allometric biomass equations, following the common approach adopted in urban forestry research [37]. Because biomass equations differ among plant types and growth environments [38], equations from areas near the study region or from regions with similar climatic conditions were preferred. Species-specific equations were applied first; when unavailable, equations for the same genus, family, or comparable growth form were substituted. Belowground carbon storage was not included, following Clark et al. [39].

The 150 quadrats contained 28 dominant woody species or species groups. Among them, 21 species used local or North China regional species-specific biomass equations, covering approximately 75% of all plant individuals. The remaining 7 ornamental shrub or less common tree species lacked local species-specific equations and were therefore estimated using genus-level or growth-form-level surrogate equations, accounting for approximately 25% of all plant individuals. Species-specific carbon content coefficients were used where possible rather than a uniform value, to improve the accuracy of species-level carbon estimates. The biomass equations and carbon content coefficients used in this study are listed in Table 2.

For each plant individual, aboveground biomass was estimated using the corresponding biomass equation based on diameter at breast height, tree height, crown width, or other required structural parameters [46,47]. The carbon storage of each individual was calculated as:

$$C_{i,t}=B_{i,t}\times CF_{i}$$

where *C_i,t_* is the carbon storage of plant individual *i* at time *t*, *B_i,t_* is the estimated aboveground biomass of plant individual *i* at time *t*, and *CF_i_* is the carbon content coefficient of species *i*.

Community-level carbon sequestration was obtained by summing the carbon storage of all individuals within each quadrat:

$$CS_{t}=\sum_{i=1}^{n}C_{i,t}$$

where *CS_t_* is the total carbon sequestration of the plant community at time *t*, and *n* is the number of plant individuals in the community.

Rather than applying the space-for-time substitution method commonly used in related studies—which does not fully account for interspecific competition and may over- or underestimate community carbon storage—this study used the dynamic succession model (Section 2.3) to simulate changes in plant structural attributes over 50 years, providing a more ecologically realistic basis for carbon sequestration estimation at each life-cycle stage.

Carbon emission factors are core parameters for quantifying greenhouse gas emissions. The IPCC carbon emission factor method was adopted: carbon emissions from energy sources such as gasoline and diesel were calculated using the carbon emission factors recommended by IPCC 2023 [48]; factors for tap water and compound fertilizer were obtained from previous studies [25]; and labor-related carbon emissions were calculated following previous research [49]. All emission factors were harmonized to a consistent carbon-unit basis of kg C per unit input (Table 3).

Data were collected through questionnaires, work-log records, and direct interviews with green-space managers, covering vehicle types, transportation distances, fuel consumption, machinery types, operation frequency, irrigation amount, fertilizer input, pesticide use, pruning frequency, litter disposal, and labor input. The calculation items for construction and maintenance emissions are shown in Table 4.

Carbon emissions from construction and maintenance were calculated using the emission factor method:

$$CE_{k}=Q_{k}\times EF_{k}$$

where *CE_k_* is the carbon emission from process *k*, *Q_k_* is the quantity of material or energy consumed, and *EF_k_* is the corresponding carbon emission factor [50].

The total carbon emission of each plant community over the 50-year life cycle was calculated as:

$$CE_{total}=CE_{construction}+CE_{maintenance}$$

where *CE_construction_* represents emissions from initial construction activities, and *CE_maintenance_* represents cumulative emissions from maintenance activities throughout the life cycle.

The net carbon balance of each plant community was calculated as:

$$NCB_{t}=CS_{t}-CE_{t}$$

where *NCB_t_* is the net carbon balance of the plant community at time *t*, *CS_t_* is carbon sequestration through aboveground biomass accumulation, and *CE_t_* is cumulative carbon emission from construction and maintenance. A positive *NCB_t_* indicates a net carbon sink, whereas a negative value indicates a net carbon source. Carbon sequestration, carbon emissions, and net carbon balance were standardized to kg·C·ha^−1^ to ensure comparability among plant communities. Annual emissions were expressed as kg·C·ha^−1^·yr^−1^ where necessary. Planting density was expressed as plants ha^−1^. Only aboveground biomass carbon was considered; belowground biomass, soil organic carbon, and litter carbon pools were not included because of data limitations and the difficulty of applying consistent root biomass equations across all species and community types.

### 2.5. Maintenance Scenario Design

**Figure 5:**
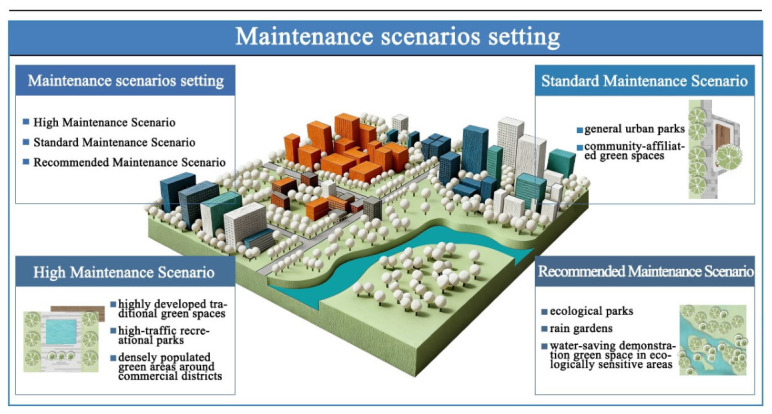
Maintenance scenarios setting.

To assess how maintenance intensity affects the carbon balance of plant communities, three maintenance scenarios were established: high-maintenance, standard-maintenance, and recommended-maintenance scenarios (Figure 5). These scenarios were defined according to local landscape maintenance regulations, management records, and interviews with green-space managers and were designed as scenario-based simulations rather than field-controlled experiments. The three scenarios differed in irrigation frequency, fertilization intensity, pesticide application, pruning frequency, litter disposal, machinery use, and labor input.

The main quantitative definitions of the three maintenance scenarios are provided in Table 5, which reports cumulative inputs per quadrat over the 50-year life cycle. The same functional unit and accounting boundary were applied across all scenarios.

The high-maintenance scenario represented intensive human intervention with frequent irrigation, regular shaping and pruning, precise fertilization, and systematic pest and disease control, typical of highly managed green spaces in urban core areas such as municipal squares, commemorative parks, high-traffic recreational parks, and green spaces around commercial districts.

The standard-maintenance scenario represented conventional management practices based on local landscape maintenance regulations and technical standards, maintaining landscape quality while controlling management costs, applicable to general urban parks and community green spaces with routine recreational and ecological functions.

The recommended-maintenance scenario followed the principles of resource-saving and low-carbon management, aiming to reduce water, fertilizer, pesticide, energy, and labor inputs by relying more on plant adaptability and community self-regulation. This scenario is more suitable for ecological parks, rain gardens, and other green spaces where low-intervention management is feasible; however, its projected carbon advantage is contingent on plant survival, health, and structural stability being maintained under reduced inputs.

### 2.6. Structural Indicators, Statistical Analysis and Uncertainty Considerations

To identify the structural factors affecting the net carbon balance of urban plant communities, a set of community-level indicators was selected, including planting density, canopy closure, tree–shrub ratio, evergreen tree ratio, Shannon–Wiener diversity index, Simpson diversity index, Patrick richness index, three-dimensional green quantity, vertical structural layers, and Pielou evenness index.

All structural indicators were standardized using Z-score transformation before statistical analysis. Pearson correlation analysis was first used to examine the relationships between community structural indicators and modeled net carbon balance. Stepwise multiple linear regression was then used to identify the dominant structural predictors, with variables selected using a forward stepwise procedure based on Akaike information criterion (AIC) minimization and *p* < 0.05. Multicollinearity was assessed using variance inflation factors (VIFs), and variables with VIF > 10 were excluded from the final model. All statistical analyses were conducted using R 4.2.

A sensitivity analysis was conducted to evaluate the influence of key maintenance inputs and model parameters on carbon emissions and modeled net carbon balance, using a ±20% perturbation approach. For maintenance-related emissions, the tested factors included irrigation, fertilizer use, pesticide use, litter disposal, labor, and pruning. For model parameters, the tested factors included kf, gm, Dm, β, kT, and k. The sensitivity index was calculated as:

$$SI=\frac{(Y_{p}-Y_{0})/Y_{0}}{(P_{p}-P_{0})/P_{0}}$$

where *SI* is the sensitivity index, *Y*_0_ is the baseline output, *Y_p_* is the output under parameter perturbation, *P*_0_ is the baseline parameter value, and *P_p_* is the perturbed parameter value. A larger absolute *SI* value indicates a stronger influence of the parameter on model outputs. The sensitivity analysis results are reported in Section 3.

Several assumptions were made to ensure consistent comparison among plant communities. The model focused on aboveground biomass carbon and excluded belowground biomass, soil organic carbon, and litter carbon dynamics. The life-cycle system boundary included major construction and maintenance processes but excluded seedling production, seedling replacement, production of gardening tools, building demolition, and maintenance after natural disasters. The 50-year simulation assumed that current maintenance technologies and carbon emission factors remained broadly stable over the modeled period. Biomass equations were selected using consistent substitution criteria across all communities, allowing comparative evaluation of relative differences in net carbon balance even where absolute estimates carry uncertainty from surrogate equations.

## 3. Results

### 3.1. Succession Model Validation

Before applying the model to the 50-year carbon source–sink simulation, an independent validation was conducted to evaluate whether the dynamic succession module could reproduce the observed long-term structural changes. The validation was based on continuous 30-year field monitoring records and park management statistics from Tianjin People’s Park. The validation dataset included 10 quadrats and 76 trees and covered the observed changes in diameter at breast height (DBH), crown width, and tree height.

**Figure 6:**
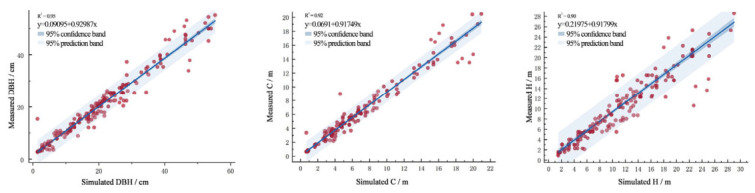
Validation of the dynamic succession model using 30-year monitoring data from Tianjin People’s Park.

The initial structural records of the validation trees were used as model inputs, and the predicted DBH, crown width, and tree height trajectories were compared with the corresponding observed values. The validation results showed that the model reproduced the observed long-term structural trends with low prediction error. The mean percentage errors (MPEs) of the main structural indicators were all below 0.5%, indicating that the simulated structural trajectories were generally consistent with the observed long-term growth records in the validation dataset (Figure 6).

This validation provides empirical support for the biological plausibility of the dynamic succession module; however, as the validation dataset was limited to 10 quadrats and 76 trees from one park and assessed structural growth rather than full life-cycle carbon balance, subsequent carbon source–sink trajectories should be interpreted as model-based scenario projections rather than deterministic predictions of actual future carbon dynamics.

### 3.2. Dynamic Outputs of Plant Community Succession Simulation

**Figure 7:**
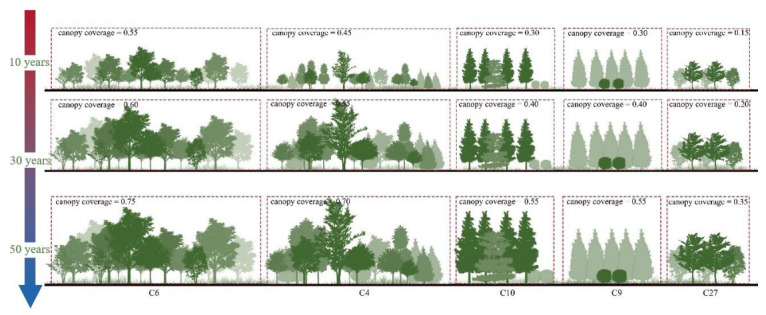
Simulation of 50-Year Growth Succession of Certain Plant Communities. (C4: Plant Community 4; C6: Plant Community 6; C9: Plant Community 9; C10: Plant Community 10; C27: Plant Community 27).

The dynamic succession module generated time-series structural trajectories for the 150 typical urban plant communities over the 50-year simulation period. These trajectories included changes in diameter at breast height (DBH), crown width, tree height, and survival status, providing the structural basis for subsequent carbon sequestration accounting. The simulated growth trajectories of DBH, crown width, and tree height for selected representative communities are shown in Figure 7.

From the perspective of individual plant growth dynamics, DBH showed a clear increasing trend over time. For example, in Community 10, the DBH of *Juniperus chinensis* increased from 4.70 cm at year 10 to 15.34 cm at year 50, with an absolute increment of 10.64 cm and a relative increase of 226.7%. In a representative broadleaf tree community 6, the DBH of *Platanus acerifolia* increased from 20.06 cm at year 10 to 62.79 cm at year 50. Compared with DBH, tree height growth was more moderate. The height of *Juniperus chinensis* in Community 10 increased from 1.10 m to 1.13 m over 50 years, whereas the height of *Platanus acerifolia* in Community 6 increased from 10.69 m to 19.05 m, while the *Fraxinus chinensis* in the same community started at 16.89 m and reached only 22.25 m after 50 years. The expansion of crown width was generally slower than DBH growth. For instance, the crown width of *Juniperus chinensis* in Community 10 increased from 0.60 m to 0.62 m, while *Platanus acerifolia* in Community 6 increased from 5.25 m to 5.43 m.

The very small height and crown-width increments of species such as Juniperus chinensis reflect the combined constraints of species-specific growth form, routine pruning, high planting density, and neighbourhood competition in managed urban settings, rather than an absence of biological growth (see Section 4.2).

DBH was the most sensitive structural indicator of long-term growth across all simulated communities, whereas tree height and crown width were more strongly constrained by species-specific growth potential and spatial competition. Across all simulated communities, tree species showed the most pronounced DBH growth, with average DBH reaching 42.3 ± 9.2 cm after 50 years. Light-demanding tree species, such as *Platanus* and *Sophora japonica*, showed stronger growth advantages. In contrast, shrubs exhibited slower DBH growth. In tree–shrub mixed communities, shrubs generally showed lower survival rates and smaller average DBH than shrubs in shrub-dominated communities, suggesting that upper-layer tree growth can suppress shrub development through light and space competition.

The variation in DBH among individuals of the same species also changed over time. In the early stage, intraspecific DBH variation was relatively narrow, and individual differences among communities showed considerable overlap. In the later stage, intraspecific differences expanded, and individuals of the same species developed more distinct structural trajectories under different community contexts. These results demonstrate that the succession module can generate differentiated long-term growth trajectories by integrating species growth potential and community-level competition effects.

### 3.3. Algorithm-Generated Carbon Sequestration Trajectories

**Figure 8:**
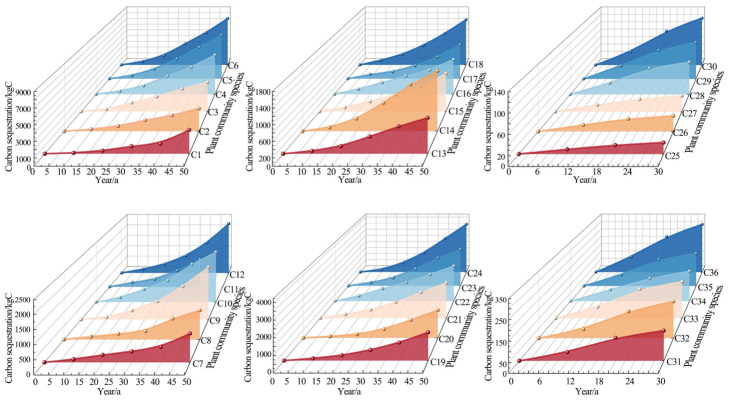
Carbon sequestration trajectories of plant communities in 50 years. (C1–C36: Plant Community 1-Plant Community 36).

Based on the structural trajectories generated by the dynamic succession model, carbon sequestration was calculated for the 150 plant communities at different life-cycle stages. This procedure transformed conventional static biomass estimation into a time-dependent carbon sequestration trajectory. Figure 8 shows the carbon sequestration of 36 representative plant communities over the 50-year period.

Community 6 had the highest gross carbon sequestration after 50 years, reaching 7882.78 kg·C·ha^−1^. This community was characterized by a closed, broadleaf, multi-layered structure, with dominant species including *Populus tomentosa*, *Fraxinus chinensis*, *Malus spectabilis*, *Lonicera japonica*, all of which are locally adapted to Tianjin and exhibit relatively strong growth and carbon accumulation potential. In contrast, Community 25 had the lowest gross carbon sequestration, with only 22.56 kg·C·ha^−1^ after 50 years. This community was dominated by a simplified single-layered boxwood shrub structure, in which low mature woody biomass, limited vertical stratification, small canopy volume, and strong pruning control collectively constrained long-term biomass accumulation.

The carbon sequestration trajectories differed substantially among community types. Tree-dominated and tree-shrub mixed communities showed continuous increases in carbon sequestration throughout the simulation period, with accumulation rates tending to accelerate as trees entered later biomass accumulation stages. Shrub-dominated communities showed rapid carbon accumulation during the early stage followed by gradual stabilization, indicating that shrubs contribute to early-stage carbon storage but their lower mature biomass limits long-term sequestration potential.

The sensitivity analysis indicated that modeled carbon sequestration was particularly sensitive to growth-related parameters: the health-response coefficient (kf, SI = 1.72) and maximum annual growth increment (gm, SI = 1.64) showed the highest sensitivity indices among all tested parameters (see Section 3.8), underscoring that long-term carbon-balance projections depend substantially on the accuracy of species-specific growth calibration.

### 3.4. Life-Cycle Carbon Emission Outputs from the LCA Module

**Figure 9:**
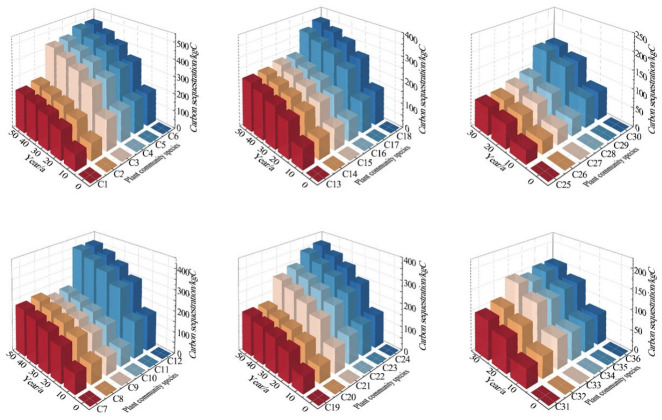
Carbon emissions of plant communities in 50 years. (C1–C36: Plant Community 1–Plant Community 36).

The LCA module quantified carbon emissions from construction and maintenance processes for the 150 plant communities. Figure 9 shows differences in life-cycle carbon emissions among 36 representative community types. The results indicate that carbon emissions varied substantially among communities, reflecting differences in community structure, planting density, species composition, and maintenance demand.

Community 4 had the highest cumulative carbon emissions, reaching 493.51 kg·C·ha^−1^. This community had a closed, multi-layered mixed structure, high canopy closure, high planting density, and a large number of plant individuals, all of which increased competition for light, water, nutrients, and growing space and thereby raised maintenance demand. In contrast, Community 33 had the lowest life-cycle carbon emissions at 91.07 kg·C·ha^−1^, characterized by an open, single-layered structure, sparse planting, and a high proportion of locally adapted species that reduced the need for intensive maintenance.

Carbon emissions originated from both construction and maintenance phases. Construction-related emissions were relatively low because construction activities occurred only once at the initial stage of the life cycle. The average construction emission was 4.87 kg·C·ha^−1^, ranging from 1.66 to 8.61 kg·C·ha^−1^. By contrast, maintenance activities occurred repeatedly throughout the life cycle and therefore contributed more substantially to cumulative emissions, with a mean annual carbon emission of 7.91 kg·C·ha^−1^.

**Figure 10:**
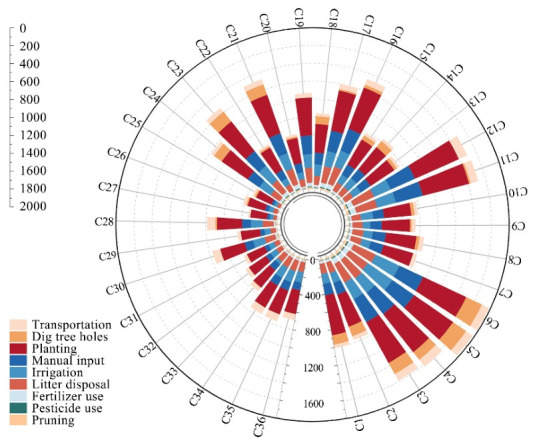
Relative contribution of different emissions sources to carbon emissions. (C1–C36: Plant Community 1–Plant Community 36).

Among all maintenance processes, fertilization, pesticide application, and irrigation were the dominant contributors to cumulative emissions across the 36 representative communities (Figure 10), indicating that resource-intensive maintenance operations constitute the primary carbon cost of urban plant community management.

### 3.5. Coupled Carbon Source–Sink Transition of Plant Communities

**Figure 11:**
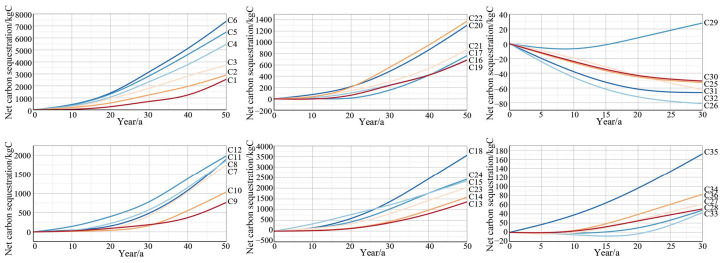
Net carbon balance and carbon source–sink transition of plant communities over 50 years. (C1–C36: Plant Community 1–Plant Community 36).

By coupling succession-driven carbon sequestration trajectories with construction and maintenance emissions, the model generated net carbon balance trajectories for each plant community. Figure 11 shows that most communities functioned as net carbon sources during the early stage because construction and maintenance emissions exceeded biomass carbon accumulation. As plant biomass increased over time, the majority of communities gradually shifted from net carbon sources to net carbon sinks. Under the baseline parameter set, 86.1% of the plant communities were projected to become net carbon sinks after 50 years; this proportion should be interpreted as a scenario-based model output under the specified system boundary, growth parameters, maintenance inputs, and emission factors, rather than a universal empirical outcome (see Section 3.8 for parameter sensitivity).

Community 6 had the highest net carbon balance after 50 years, reaching 3186.08 kg·C·ha^−1^. This community had a closed, multi-layered broadleaf structure with moderate planting density and a high proportion of species with strong carbon sequestration capacity. Its relatively low maintenance emissions also contributed to its favorable net carbon balance. In contrast, Community 25 had the lowest net carbon balance, at −81.21 kg·C·ha^−1^. This community had weak biomass accumulation and remained unable to fully offset construction and maintenance emissions during the 50-year simulation.

These results demonstrate that net carbon balance depends on the coupling between biomass accumulation and life-cycle emissions: communities with structurally complex but stable configurations, locally adapted species, and moderate maintenance demand were more consistently projected to become strong long-term carbon sinks.

### 3.6. Structural Drivers Identified by the Model

**Figure 12:**
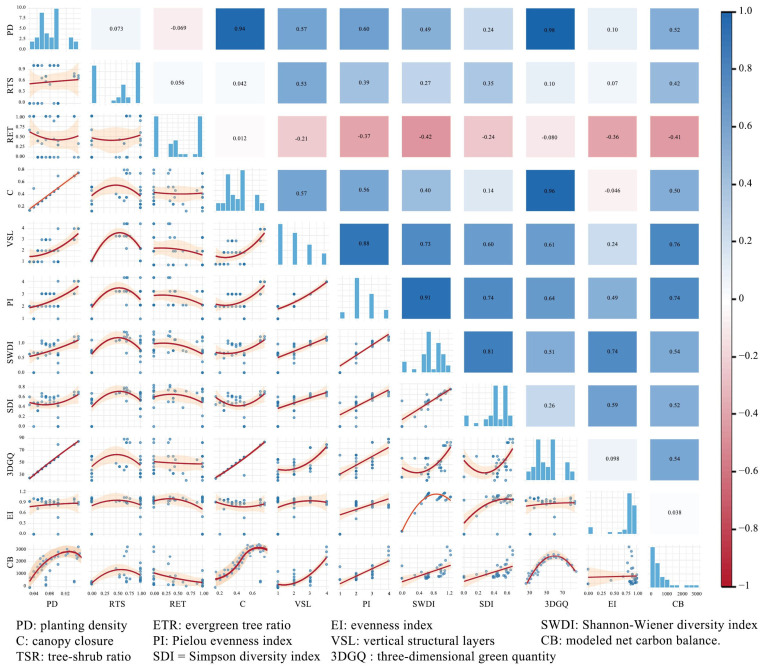
Analysis of structural factors influencing net carbon sequestration in plant communities.

To identify structural factors associated with model-derived net carbon balance, community-level indicators were standardized and analyzed using correlation and regression methods. The selected indicators included planting density, canopy closure, tree–shrub ratio, evergreen tree ratio, diversity indices, species richness, three-dimensional green quantity, vertical structural layers, and evenness. The results are shown in Figure 12.

Vertical structure, canopy closure, species richness, the tree ratio, density, three-dimensional green quantity, and the diversity index were positively associated with carbon balance, Whereas the evergreen ratio showed a negative association. Among all structural indicators, vertical structure showed the strongest positive correlation (r = 0.76): Community 6, with a vertical structure coefficient of 4, achieved a net carbon balance of 3186.09 kg·C·ha^−1^, whereas Community 35, with a vertical structure coefficient of 1, had a much lower net carbon balance of 172.16 kg·C·ha^−1^.

Species richness also showed a strong positive association (r = 0.74), with each additional species associated with an average increase of approximately 322 kg·C·ha^−1^ in net carbon balance. Three-dimensional green quantity and canopy closure had moderate positive associations (r = 0.54 and r = 0.52, respectively). The effect of canopy closure was non-linear: net carbon balance increased when canopy closure was below 0.75 but tended to decline beyond this threshold, suggesting an optimal operating range of 0.50–0.75. Evergreen ratio showed a weaker negative association (r = −0.41).

To address potential collinearity among structural indicators, standardized multiple regression analysis was conducted after Z-score transformation of all predictors. Variance inflation factors (VIFs) were used to evaluate multicollinearity, and variables with VIF > 10 were excluded from the final regression model. The final model retained vertical structural complexity and species richness as the dominant positive predictors of modeled net carbon balance.

Overall, the structural driver analysis showed that the model-derived net carbon balance was most strongly associated with vertical structural complexity and species richness. This indicates that the proposed framework can not only simulate carbon source–sink trajectories, but also identify key structural parameters linked to long-term carbon performance.

### 3.7. Carbon Balance Trajectories Under Different Maintenance Scenarios

**Figure 13:**
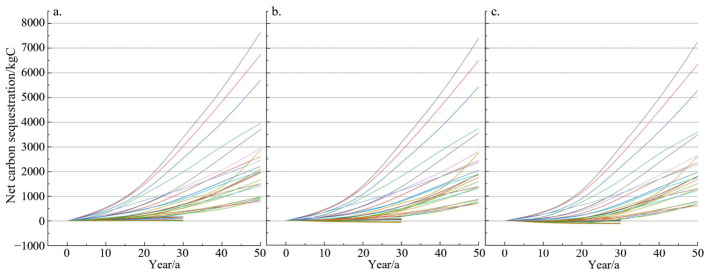
Carbon balance curves of plant communities under different maintenance scenarios: (**a**) recommended-maintenance scenario; (**b**) standard-maintenance scenario; (**c**) high-maintenance scenario.

Figure 13 compares carbon balance trajectories under three maintenance scenarios: recommended-maintenance, standard-maintenance, and high-maintenance.

Under the high-maintenance scenario, many communities showed strong fluctuations in net carbon balance during the first 20 years, primarily because high construction and maintenance inputs during the early establishment stage exceeded vegetation carbon sequestration. Although intensive maintenance improved growth conditions for some communities, additional emissions from irrigation, fertilization, pesticide application, pruning, and labor input offset part of the increased carbon sequestration, leaving more persistent carbon-source communities by the end of the simulation.

Under the standard-maintenance scenario, most communities initially acted as carbon sources but gradually shifted toward carbon sinks over time. By the end of the 50-year life cycle, only a small number of communities remained weak carbon sources, while most had become stable carbon sinks.

Under the recommended-maintenance scenario, most communities shifted more rapidly toward carbon sinks than under the other two scenarios. Reduced water, fertilizer, pesticide, energy, and labor inputs lowered maintenance-related carbon emissions and improved overall net carbon benefits.

Comparative analysis of the three scenarios showed that higher maintenance intensity did not necessarily produce better carbon outcomes: the recommended-maintenance scenario consistently achieved a more favorable balance between carbon sequestration and emission reduction than the high-maintenance scenario across the majority of community types.

### 3.8. Sensitivity Analysis of Model Parameters and Maintenance Inputs

To evaluate the uncertainty of the simulation results, a ±20% sensitivity analysis was conducted for key maintenance inputs and model parameters. For maintenance-related emissions, the tested factors included irrigation, fertilizer use, pesticide use, litter disposal, labor, and pruning. For model parameters, the tested factors included kf, gm, Dm, β, kT, and k.

Among maintenance inputs, irrigation showed the highest sensitivity index (SI = 1.92), followed by pesticide use (SI = 1.46), labor input (SI = 1.27), litter disposal (SI = 1.00), fertilizer use (SI = 0.92), and pruning (SI = 0.79). These results indicate that uncertainty in irrigation and pesticide inputs can substantially affect life-cycle carbon emission estimates.

Among model parameters, the health-response coefficient kf showed the highest sensitivity index (SI = 1.72), followed by the maximum annual growth increment gm (SI = 1.64), maximum DBH parameter Dm (SI = 1.21), neighborhood competition coefficient β (SI = 0.63), temperature sensitivity coefficient kT (SI = 0.36), and radiation-response parameter k (SI = 0.11). These results indicate that modeled net carbon balance is most sensitive to growth-response and maximum growth-potential parameters.

Overall, the sensitivity analysis confirms that both maintenance inputs and biological growth parameters introduce meaningful uncertainty into projected carbon source–sink transitions, and that accurate species-specific calibration of kf and gm is essential for improving the reliability of long-term carbon-balance simulations.

## 4. Discussion

### 4.1. Ecological Interpretation of the Dynamic Life-Cycle Simulation Framework

The primary contribution of the proposed framework is the integration of processes that are typically treated separately in urban green-space carbon studies—plant structural growth, environmental suitability, neighbourhood competition, aboveground biomass carbon accumulation, and construction- and maintenance-related emissions—within a single consistent life-cycle boundary. This integration shifts carbon assessment from a static snapshot of biomass stocks to a dynamic comparison of net carbon balance trajectories, allowing different plant community structures and maintenance regimes to be evaluated under equivalent assumptions.

A key implication of this coupled approach is that carbon source–sink status should not be treated as a fixed attribute of an urban plant community. In the early establishment stage, construction and maintenance inputs may exceed aboveground biomass carbon accumulation, causing communities to function as modeled net carbon sources. As vegetation develops and biomass accumulates, communities may gradually shift toward modeled net carbon sinks. This dynamic perspective helps explain why static carbon-stock accounting may overestimate or underestimate the long-term carbon benefit of designed urban plant communities. Equally important, a community with high gross carbon sequestration does not necessarily produce high net carbon benefit if it requires intensive irrigation, fertilization, pesticide application, pruning, or labor input; conversely, a structurally stable community composed of locally adapted species may achieve a more favorable modeled net carbon balance when maintenance demand remains moderate. This interpretation is consistent with previous urban green-space LCA studies showing that operational emissions can substantially delay the time required for urban vegetation projects to offset establishment and maintenance emissions [51,52,53].

For planting design and management, the practical value of the proposed framework lies in its capacity to compare design and management alternatives under specified structural and maintenance assumptions, rather than to claim absolute long-term carbon outcomes. The model outputs are most appropriately used for identifying community configurations and maintenance regimes that are projected to accelerate the transition from carbon source to carbon sink.

### 4.2. Structural Mechanisms Underlying Model-Identified Carbon Balance Patterns

The model-derived results showed that plant community structure strongly influenced long-term net carbon balance. Community type was a primary determinant of carbon sequestration trajectories: tree-dominated and multi-layered tree-shrub communities accumulated biomass more continuously over time, whereas shrub-dominated communities showed early-stage accumulation followed by stabilization at lower levels, consistent with the broader finding that mixed broad-leaved, vertically complex communities tend to store more carbon than simplified single-layer or shrub-dominated communities [7]. Among all structural indicators, vertical structural layers showed the strongest positive association with net carbon balance, consistent with Liu et al. [54], who emphasized the role of vertical stratification in enhancing the carbon sink function of urban vegetation. A multi-layered structure intercepts sunlight at different heights, increases photosynthetic surface area, and improves the spatial use efficiency of urban green spaces, allowing the community to accumulate biomass more continuously over time.

Species richness was also strongly associated with net carbon balance, with each additional species associated with an average increase of approximately 322 kg·C·ha^−1^ in net carbon balance. In species-rich communities, differences in crown architecture, shade tolerance, root distribution, phenology, and growth rate may promote resource partitioning, functional complementarity, and niche differentiation, thereby improving overall resource-use efficiency and long-term carbon accumulation [55,56]. However, species richness may covary with vertical stratification, planting density, canopy closure, and management history, and these associations should not be interpreted as direct causal effects.

Canopy closure and planting density also played important roles, but their effects were non-linear. A moderate increase in canopy closure can enhance light interception, increase leaf area, and improve community photosynthetic capacity [57,58]. However, excessive canopy closure or density can restrict individual growing space, intensify intraspecific and interspecific competition, and trigger self-thinning effects [59]. Excessive canopy overlap may also reduce light availability for lower leaves, suppress understory growth, and increase the risk of pests and diseases [14]. Net carbon balance increased when canopy closure was below 0.75 but tended to decline beyond this threshold, consistent with Yang et al., who found an inverted U-shaped relationship between canopy closure and carbon sink capacity in urban green spaces in Nanjing, with optimal carbon balance occurring when canopy closure ranged from 0.50 to 0.75 [60].

The tree–shrub ratio and evergreen tree ratio further affected net carbon balance by changing community composition and resource competition. Net carbon sequestration tended to reach a threshold when the tree–shrub ratio approached 0.7 and declined beyond this point, broadly consistent with Guo et al., who reported higher net carbon sequestration when the tree–shrub ratio ranged from 0.5 to 0.7 [61], suggesting that increasing the proportion of trees can improve carbon sequestration but excessive dominance of the tree layer may intensify shading and suppress shrub-layer growth.

The negative relationship between evergreen tree ratio and net carbon balance may reflect scale effects. At the individual scale, evergreen species benefit from longer photosynthetic duration, and Ye et al. found the life-cycle carbon balance of evergreen and deciduous woody species to be approximately equivalent [62]. At the community scale, however, a high evergreen proportion may alter canopy light distribution, increase water competition, and reduce the growth of other layers [63], thereby affecting net carbon balance.

The high-performing communities identified by the model were generally characterized by locally adapted species, multi-layered structures, and relatively moderate maintenance demand. Communities containing species such as *Populus tomentosa*, *Salix babylonica*, *Fraxinus chinensis*, *Malus spectabilis*, and *Lonicera japonica* showed stronger net carbon sequestration. These species are relatively well adapted to the climatic and soil conditions of Tianjin, including cold stress, salinity, alkalinity, and poor soil fertility, reducing the need for irrigation, fertilization, and pest control while supporting stable biomass accumulation, consistent with Joswig et al.’s emphasis on the importance of selecting species suited to local environmental conditions [64].

Taken together, these structural patterns suggest that low-carbon planting design should optimize vertical complementarity, species compatibility, local adaptability, and maintenance demand, rather than simply maximizing biomass or planting density.

### 4.3. Maintenance Emissions and Scenario Implications

The results of this study show that maintenance-related emissions are a critical component of plant community life-cycle carbon balance. Urban plant communities are not only biological carbon sinks, but also managed systems that require continuous inputs of water, fertilizer, pesticide, machinery, transportation, and labor. If these emissions are ignored, the net carbon benefit of urban green spaces may be overestimated. This finding is consistent with Nowak et al., who reported that construction and maintenance measures could have an even greater influence on net carbon sequestration than community structure in some urban tree systems [51].

Among all maintenance processes, fertilization was the dominant contributor to carbon emissions, followed by pesticide application and irrigation, a pattern broadly consistent with previous urban green-space LCA studies reporting that resource-intensive maintenance operations—particularly irrigation, fertilizer input, and agrochemical use—account for a large proportion of operational emissions [25,53]. This pattern differs from Kim et al., who found that pruning accounted for the largest proportion of maintenance emissions for Korean street trees [52]. The difference may reflect the distinct management context of park plant communities relative to street trees: street trees are strongly constrained by road safety and traffic clearance, making pruning a dominant maintenance activity, whereas park communities with more diverse species composition and complex structures require regular fertilization, irrigation, and pest control to maintain landscape quality and ecological stability under challenging soil and climatic conditions.

The dominance of fertilization as an emission source is consistent with Xiao et al., who reported that fertilizer-related CO_2_ emissions accounted for 46.67% of maintenance-stage emissions in Lujiazui Central Green Space [53]. Chemical fertilizers and pesticides are associated with high energy consumption and substantial upstream emissions: the production of 1 t of compound fertilizer can release 308.53 kg of CO_2_, while the production of 1 t of chemical pesticide can release 7.73 t of CO_2_ [65]. Therefore, reducing dependence on chemical inputs—through organic fertilizer substitution, biological pest control, and adaptive irrigation—represents a particularly effective lever for lowering the life-cycle carbon cost of urban plant community management.

Labor-related carbon emissions were also included in this study. Although labor factors have been incorporated into LCA studies in fields such as architecture and medical care [66,67,68], they are still rarely considered in plant community research. This study found that 15.24% of the carbon emissions generated during construction and maintenance were attributable to anthropogenic activities, suggesting that future LCA studies should explicitly define whether labor-related emissions are included within the system boundary.

The maintenance scenario analysis further demonstrates that higher maintenance intensity does not necessarily lead to better carbon outcomes. High-maintenance management may improve growth conditions by reducing water stress, nutrient limitation, and pest damage, but these benefits can be offset by additional emissions from irrigation, fertilization, pesticide application, pruning, machinery use, and labor input. However, the recommended-maintenance scenario should not be interpreted as the absence of management or as universally applicable low-input management: its carbon advantage depends entirely on whether plant survival, health, and structural stability can be maintained under reduced inputs. If reduced maintenance leads to water stress, pest outbreaks, increased mortality, or lower biomass accumulation, the projected carbon benefit of this scenario may be weakened or reversed. Low-carbon maintenance should therefore match intervention intensity with community structure, plant adaptability, successional stage, and site-specific stress conditions, rather than simply minimizing all inputs.

From a model application perspective, the scenario analysis demonstrates that the proposed framework can be used to compare alternative maintenance strategies before implementation, helping to identify management pathways that accelerate the transition from carbon source to carbon sink—a capability that is particularly useful when aesthetic quality, ecological function, maintenance cost, and carbon performance must be balanced simultaneously.

### 4.4. Implications for Low-Carbon Planting Design and Model Application

The transition from scientific assessment to practical application is essential for realizing the carbon mitigation potential of urban green spaces. The proposed model provides a basis for translating carbon accounting results into planting design strategies because it links plant community structure, succession dynamics, maintenance demand, and net carbon balance. Therefore, the design recommendations derived from this study should be understood as model-informed strategies rather than simple empirical rules.

For species selection, priority should be given to locally adapted species with strong growth potential, stress tolerance, and relatively low maintenance demand. Native or locally adapted species can better cope with local climatic and soil constraints and may require less irrigation, fertilization, and pest control. In Tianjin, species with cold tolerance, salt-alkali tolerance, and adaptability to poor soil conditions are particularly valuable. Species selection should also consider compatibility among species, crown architecture, shade tolerance, root distribution, and growth rate, as these traits affect neighbourhood competition and long-term community stability.

For community structure, the results indicate that multi-layered communities generally have stronger long-term carbon benefits than simplified single-layer structures. Tree–shrub–herb combinations can improve vertical space use, increase the photosynthetic surface area, and enhance biomass accumulation. However, structural complexity should not be equated with excessive density: a moderate canopy closure within the 0.50–0.75 range, balanced tree–shrub composition approaching a ratio of 0.5–0.7, and reasonable planting density are more suitable for low-carbon plant community design than simply maximizing the number of layers or individuals.

For maintenance management, fertilizer and pesticide use should be carefully controlled, and organic fertilizers, biological pest control, recycled water, adaptive irrigation, and reduced pruning frequency should be encouraged where appropriate. Maintenance should also be differentiated by community development stage: young communities may require temporary establishment inputs, while structurally stable mature communities can gradually shift toward lower-input adaptive management.

**Figure 14:**
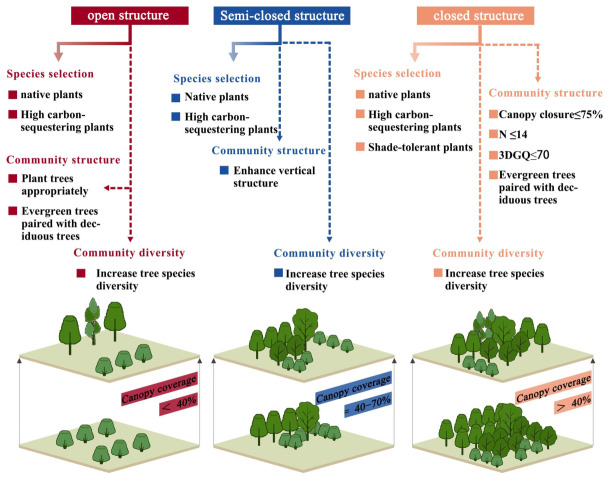
Planting design strategies for plant communities.

Based on the model-identified structural characteristics and carbon balance patterns, this study proposes differentiated planting design strategies for open, semi-closed, and closed plant communities (Figure 14). For open communities, native plants and high-carbon-sequestration tree species should be prioritized, with reasonable tree arrangement and a mixture of evergreen and deciduous species to improve ecological function and landscape sustainability. For semi-closed communities, vertical structural layers should be strengthened through tree–shrub–herb combinations to improve spatial resource use and carbon sequestration potential. For closed communities, shade-tolerant species should be introduced, canopy closure should be controlled within a suitable range, and excessive density should be avoided to maintain system health and functional balance.

### 4.5. Model Assumptions, Uncertainty, and Transferability

Several assumptions and uncertainties should be considered when interpreting the results of this study. The dynamic succession model integrates multiple parameter sets—environmental suitability coefficients, neighbourhood competition weights, Plant Health Index coupling parameters, growth response coefficients, mortality thresholds, biomass equations, carbon content coefficients, emission factors, and maintenance inputs—each of which represents a simplification of complex ecological processes. The sensitivity analysis (Section 3.8) quantifies the influence of key parameters and maintenance inputs on model outputs, and the reported results should be interpreted within these uncertainty bounds.

The 50-year simulation assumes that current maintenance technologies, emission factors, and management rules remain broadly comparable over the modeled period. Future changes in irrigation systems, fertilizer production, machinery efficiency, labor organization, and low-carbon maintenance technologies could alter life-cycle emissions, and changes in planting design standards or municipal maintenance policies may affect the relationship between maintenance intensity, plant health, and carbon performance.

The carbon accounting boundary was limited to aboveground vegetation carbon balance. Belowground biomass, soil organic carbon, litter carbon, and dead organic matter were not included because consistent species-specific and community-scale data were unavailable. This exclusion means that the results represent aboveground vegetation carbon balance rather than total ecosystem carbon balance; soil carbon and belowground biomass may represent substantial carbon pools in urban green spaces, and their omission may underestimate total carbon storage while potentially altering the absolute timing of modeled source–sink transitions. Future studies should integrate aboveground biomass, belowground biomass, soil carbon, litter, and dead organic matter to provide a more complete ecosystem-level carbon assessment.

The transferability of the model beyond Tianjin should be considered carefully. The general framework can be applied to other cities because it uses common structural variables, biomass accounting logic, and life-cycle emission accounting. However, climate conditions, soil properties, species pools, growth rates, irrigation demand, maintenance standards, and emission factors differ substantially among cities and regions, and parameter values should not be transferred directly without local calibration.

The present simulation did not explicitly incorporate future climate change. In northern Chinese cities such as Tianjin, future warming and increased climate variability may increase irrigation demand for stress-sensitive species, potentially raising both maintenance emissions and plant mortality rates. Future model development should incorporate climate-change scenarios to evaluate how altered environmental conditions may affect long-term carbon source–sink trajectories, particularly for communities near the thermal tolerance boundaries of their constituent species.

Uncertainty may also arise from the use of surrogate biomass equations. Species-specific biomass equations were used where available, covering approximately 75% of all plant individuals; genus-, family-, or growth-form-level equations were adopted for the remaining 25%, primarily ornamental shrubs or less common tree species. This substitution is common in urban forestry carbon studies but may introduce uncertainty into absolute biomass and carbon estimates. Future studies should develop locally calibrated biomass equations for common urban ornamental species to improve the accuracy of community-scale carbon accounting.

Overall, the proposed model provides a useful framework for comparing the relative long-term carbon performance of different urban plant community configurations and maintenance scenarios. The results should be interpreted as scenario-based projections of aboveground vegetation carbon balance under specified structural, growth, and maintenance assumptions, most appropriately applied to comparative evaluation of planting design and management alternatives rather than as deterministic predictions of ecosystem-level carbon dynamics.

## 5. Conclusions

This study developed a dynamic succession-based life-cycle simulation model to project carbon source–sink transitions in urban plant communities. By integrating these coupled ecological and accounting processes within a consistent life-cycle boundary, the model provides a process-informed framework for comparing the modeled long-term net carbon balance of different urban plant community configurations. The results are most appropriately applied to the comparative evaluation of planting design and management alternatives rather than as deterministic predictions of ecosystem-level carbon dynamics.

Most communities were projected to act as net carbon sources during the early stage of the life cycle, when construction and maintenance emissions exceeded aboveground biomass accumulation. As simulated biomass accumulated, most communities gradually shifted toward modeled net carbon sinks. Under the baseline parameter set, 86.1% of the studied communities were projected to become net carbon sinks after 50 years—a proportion that should be interpreted as a scenario-based model output under specified structural, growth, and maintenance assumptions, rather than an empirical long-term observation or a universal outcome.

Plant community structure and maintenance intensity jointly influenced modeled net carbon balance. Among structural indicators, vertical structural complexity and species richness were identified as the strongest positive predictors, followed by three-dimensional green quantity and canopy closure. Among maintenance processes, fertilization, pesticide application, and irrigation were the dominant contributors to life-cycle emissions and substantially affected the timing and magnitude of carbon source–sink transitions. Across maintenance scenarios, resource-saving maintenance consistently improved modeled net carbon balance, but only when reduced inputs did not compromise plant health, survival, or biomass accumulation.

These findings collectively suggest that low-carbon urban planting design should emphasize locally adapted species, multi-layered structures, moderate canopy closure, species compatibility, and adaptive maintenance, rather than simply maximizing planting density or minimizing management inputs. The results represent modeled projections of aboveground vegetation carbon balance and do not account for belowground biomass, soil organic carbon, litter carbon, or dead organic matter; their exclusion may underestimate total ecosystem carbon storage and affect the absolute timing of projected source–sink transitions. Future studies should address these limitations by incorporating complete ecosystem carbon pools, locally calibrated biomass equations, broader long-term validation datasets spanning multiple cities and green-space types, and explicit climate-change scenarios, thereby improving the robustness and transferability of the proposed framework as a planning tool for low-carbon urban green infrastructure.

## Figures and Tables

**Table 1:** Key parameters used in the dynamic succession model.

Parameter	Values	Sources and Determination
kT	Broad-leaved species: 0.035–0.048; coniferous species: 0.045–0.060	Literature on plant temperature response [30,31,32] and field-measurement calibration
β	0.0012	Field-of-neighborhood competition models [33,34] and field-measurement calibration
α	0.85	Individual-based plant growth models [29] and field-measurement calibration
wT	Strong thermal adaptability: 0.25; moderate thermal adaptability: 0.55; poor thermal adaptability: 0.85	Literature on environmental stress weighting [29,35] and field-measurement calibration
kf	Fast-growing trees: 0.60–0.75; slow-growing shrubs: 0.30–0.50	Observed annual growth increments and literature on plant growth response [29,36]

**Table 2:** The carbon content and Biomass equation of different plants.

Species	Biomass Equation	Carbon Content	Reference
*Platanaceae*	B = 0.0690(D^2^H)^0.9133^	0.500	[40]
*Fraxinus chinensis*	B = 2.1893 + 0.032949D^2^H	0.470	[40]
*Salix babylonica*	B = 0.1368D^2.408^	0.465	[40]
*Styphnolobium japonicum*	B = 0.714 + 0.03(D^2^H)	0.502	[40]
*Pinus tabuliformis*	B = 0.274D^1.898^ + 0.001D^3.284^ + 0.009D^2.433^	0.518	[41]
*Ailanthus altissima*	B = 0.241D^2.046^	0.502	[41]
*Pinus bungeana*	LgB = 2.311 + 2.154lgD − 1.593 + 4.34lgD − 1.851 + 3.934lgD	0.496	[42]
*Rhus typhina*	B = 0.11D^2.3^H^0.6^	0.477	[41]
*Populus tomentosa*	B = 0.015(D^2^H)^1.032^	0.460	[41]
*Juniperus chinensis*	B = 0.067D^2.5^	0.474	[41]
*Koelreuteria paniculata*	B = 0.915 + 0.100D^2^H	0.477	[41]
*Ulmus pumila*	B = 0.1458(D^2^H)^0.8191^	0.510	[41]
*Robinia pseudoacacia*	B = 0.0768D^2.15^H^0.5^	0.465	[43]
*Syringa oblata*	B = 0.073(D^2^H)^0.76^	0.430	[43]
*Malus spectabilis*	B = 0.0638D^2^ − 0.0639D + 2.1647	0.460	[41]
*Prunus cerasifera*	B = 0.073D^1.989^	0.460	[41]
*Buxus sinica*	B = 0.12D^2.4^	0.475	[44]
*Ligustrum quihoui*	B = 14.646C^1.164^	0.500	[44]
*Lonicera japonica*	B = 0.365(D^2^H)^0.56^	0.480	[45]
*Hibiscus syriacus*	B = 2.958(D^2^H)^0.607^	0.470	[44]
*Juniperus procumbens*	B = 0.067D^2.5^	0.474	[44]

**Table 3:** The coefficients of carbon emissions.

General Category	Inputs	Emission Coefficient	Units	Reference
Energy	Diesel	0.728	kg C·L^−1^	[48]
	Gasoline	0.605	kg C·L^−1^	[48]
	Electricity	0.192	kg C·kwh^−1^	[48]
Resource	Tap water	0.248	kg C·m^−3^	[25]
	Compound fertilizer	0.455	kg C·kg^−1^	[25]
	Insecticide	1.391	kg C·kg^−1^	[25]
	Disinfectant	1.391	kg C·kg^−1^	[25]
	Human resources	0.030	kg C·h^−1^	[49]

**Table 4:** The calculation content of construction and maintenance.

General Category	Process	Calculation Range	Calculation Content
Construction	Dig tree holes	Excavator	Fuel consumption
	Delivery of trees	Vehicle	Transport mileage
	Planting	Equipment	Fuel consumption
Maintenance	Irrigation	Water production consumption	Quantity of water
	Pruning	Equipment	Fuel consumption
	Fertilizer use	Fertilizer consumption	Quantity of fertilizer
	Pesticide use	Pesticide consumption	Quantity of pesticide
	Litter disposal	Delivery of litter	Fuel consumption

**Table 5:** Quantitative definitions of maintenance scenarios.

Maintenance Scenario	Irrigation Amount (L)	Fertilizer Input (kg)	Pesticide Amount (L)	Pruning Frequency (Times)
Recommended-maintenance	59.54	34.24	4.61	150
Standard-maintenance	170.12	114.125	18.44	1000
High-maintenance	306.22	228.25	27.66	2500

## Data Availability

Data is contained within the article. The original contributions presented in this study are included in the article. Further inquiries can be directed to the corresponding author.

## Abbreviations

The following abbreviations are used in this manuscript:

LCA	Life Cycle Assessment
UGS	Urban Green Space
DBH	Diameter at Breast Height
3DGQ	Three-dimensional Green Quantity
VSL	Vertical Structural Layer
PD	Planting Density
C	Canopy Coverage
RTS	The Ratio of Tree-shrub
RET	The Ratio of Evergreen Tree
SWDI	Shannon–Wiener Diversity Index
SDI	Simpson’s Diversity Index
PRI	Patrick Richness Index
PI	Pielou’s Evenness Index
IPCC	Intergovernmental Panel on Climate Change
FON	Field of Neighbourhood
CO_2_	Carbon Dioxide
kgC/ha^2^	Kilogram Carbon per Hectare
